# CCR5Δ32 Genotypes in a German HIV-1 Seroconverter Cohort and Report of HIV-1 Infection in a CCR5Δ32 Homozygous Individual

**DOI:** 10.1371/journal.pone.0002747

**Published:** 2008-07-23

**Authors:** Djin-Ye Oh, Heiko Jessen, Claudia Kücherer, Konrad Neumann, Nari Oh, Gabriele Poggensee, Barbara Bartmeyer, Arne Jessen, Axel Pruss, Ralf R. Schumann, Osamah Hamouda

**Affiliations:** 1 Institute of Microbiology and Hygiene, Charité University Medical Center, Berlin, Germany; 2 Gemeinschaftspraxis Jessen-Jessen-Stein, Berlin, Germany; 3 Robert Koch-Institute, Berlin, Germany; 4 Department of Medical Biometry and Clinical Epidemiology, Charité University Medical Center, Berlin, Germany; 5 Institute of Transfusion Medicine, Charité University Medical Center, Berlin, Germany; National AIDS Research Institute, India

## Abstract

**Background:**

Homozygosity (Δ32/Δ32) for the 32 bp deletion in the chemokine receptor 5 (CCR5) gene is associated with strong resistance against HIV infection. Heterozygosity is associated with protection of HIV-1 disease progression.

**Methodology/Principal Findings:**

We genotyped a population of 737 HIV-positive adults and 463 healthy controls for the CCR5Δ32 deletion and found heterozygous frequencies of 16.2% (HIV-negative) and 17.5% (HIV-positive) among Caucasian individuals. Analysis of CCR5Δ32 influence on disease progression showed notably lower viral setpoints and a longer time to a CD4 count of <200 µl^−1^ in seroconverters heterozygous for the deletion. Furthermore, we identified one HIV-positive man homozygous for the Δ32 deletion.

**Conclusions/Significance:**

The protective effect of CCR5 Δ32 heterozygosity is confimed in a large cohort of German seroconverters. The HIV-infected CCR5 Δ32 homozygous individual, however, displays extremely rapid disease progression. This is the 12th case of HIV-infection in this genotype described worldwide.

## Introduction

Chemokine receptor-5 (CCR5) is of major importance for the transmission of HIV-1 (reviewed in [1], [2]). CCR5Δ32 is a deletion resulting in a defective phenotype of this receptor, and the first host polymorphism with an effect on HIV-1 disease demonstrated: CCR5Δ32 homozygous individuals are potently protected against HIV-1 infection [3]–[6]. CCR5Δ32 heterozygosity has been shown to be significantly associated with slower HIV-1 disease progression in several study cohorts [3], [7]–[11]. Whereas other human mutations have been demonstrated since then to also affect the HIV disease progression rate [12]–[15], CCR5Δ32 is still regarded as the strongest genetic factor of HIV resistance of the host known to date [2], [16]. HIV-1 infection in CCR5Δ32 homozygotes is exceedingly rare, with a total of eleven cases reported worldwide so far [17]–[26].

The objective of this study was to assess the influence of the CCR5Δ32 deletion on susceptibility to, and progression of HIV infection in a large German study population. A study group of 737 well characterized HIV-positive patients, including 499 seroconverters, and 463 HIV-negative controls, was examined for presence of the deletion allele. Our results confirm the previously conveyed CCR5Δ32 associations. Furthermore, we here report one HIV-infected male displaying homozygosity for the mutation. This individual represents the 12th HIV case with the homozygous mutant (Δ32/Δ32) genotype known in the world.

## Results

### Distribution of CCR5Δ32 genotypes in the study cohort and identification of an HIV-1 infected individual homozygous for the CCR5Δ32 deletion

In order to examine the association of the CCR5Δ32 deletion with HIV-1 susceptibility in a German study cohort for the first time, its presence was assessed in a total of 737 seropositive patients and 463 seronegative controls. Among the African study participants, the Δ32 variant was detected in only one control subject, who was heterozygous. In a total of 1139 Caucasians, 16.7% were CCR5Δ32 heterozygous (+/Δ32) and 0.2% were homozygous (Δ32/Δ32), and genotypes were distributed similarly between cases and controls: Among the seropositive study subjects, 83.7% displayed the common CCR5^+/+^ genotype, whereas 16.2% were heterozygous for the deletion allele (+/Δ32). Of the seronegative controls, the CCR5^+/+^ genotype was found in 82.2%, while 17.5% were heterozygous. No significant association of CCR5Δ32 state with serostatus was found, regardless of whether analysis was performed by genotype frequencies (Table 1) or by allele frequencies (data not shown).

**Table 1:** CCR5Δ32 genotype distribution in HIV-positive and HIV-negative study subjects

	HIV-positive	HIV-negative	
	number ( % )		P-value^1^
Caucasian			
+ / +	595 (83.7)	352 (82.2)	
+ / Δ32	115 (16.2)	75(17.5)	0.78
Δ32 / Δ32	1 (0.1)	1 (0.2)	
African			
+ / +	35 (100.0)	25 (96.2)	
+ / Δ32	0	1 (3.8)	0.24
Δ32 / Δ32	0	0	

1 2×2 or 3×2 χ2 comparisons, depending on the presence or absence of heterozygous and mutant homozygotes in the respective subgroup. Comparisons were conducted separately according to ethnicity

Of particular note, one (0.1%) HIV-1 infected individual was found to be homozygous for the CCR5Δ32 allele. The Δ32/Δ32 genotype is generally regarded to render almost complete resistance against HIV-infection.

### Association of CCR5Δ32 genotype with HIV-1 disease progression in Caucasian seroconverters

**Figure 1:**
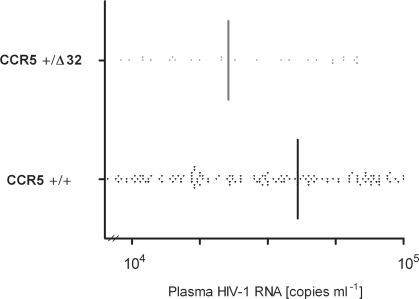
Relation of CCR5Δ32 genotype with viral setpoints in 319 Caucasian seroconverters. Scattered dots represent individual viral setpoints (see Material and Methods for a definition), whereas vertical lines demarcate the median in each genetic group.

**Figure 2:**
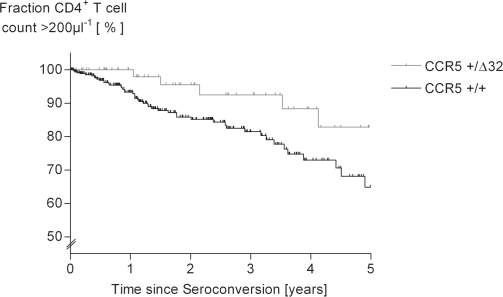
Kaplan Meier Analysis of 506 seroconverters showing a relation of CCR5Δ32 genotype with a CD4^+^ T-cell count <200 µl^−1^. A CD4^+^ T-cell count <200 µl-1 was documented for n = 57 of 422 individuals homozygous for the wildtype CCR5 genotype, and for n = 9 out of 84 CCR5Δ32 heterozygotes. Data censoring was due to introduction of antiretroviral therapy before a CD4 cell count of 200 µl^−1^ was reached. The difference between both groups implied by the Kaplan-Meier diagram is reflected in a statistical trend (p = 0.1 as calculated by the Log-Rank test).

To evaluate the impact of CCR5Δ32 on the progression of HIV-1 disease in the study cohort, the strength of the association between heterozygosity and viral load setpoints was assessed. This revealed a trend towards decreased setpoints in heterozygous seroconverters as compared to those with the common CCR5^+/+^ genotype without reaching statistical signifcance, however (p = 0.1; Fig. 1). A protective effect of CCR5Δ32 heterozygosity became also apparent in Kaplan-Meier analysis of 496 therapy-naïve seroconverters demonstrating that mutation carriers reached the study endpoint of <200 µl-1 CD4^+^ T-cells later than carriers of the common gene variant (p = 0.1; Fig. 2).

### Characteristics of an HIV-1 positive individual homozygous for the CCR5Δ32 deletion

**Figure 3:**
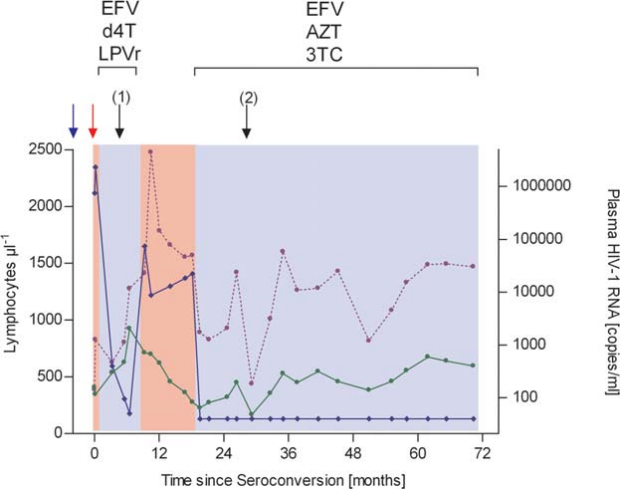
Clinical, immunological and viral load characteristics in an HIV-infected patient homozygous for the CCR5Δ32 deletion (patient#12). Antiretroviral therapy was initiated twice, eight days after diagnosis with efavirenz (EFV), stavudine (d4T) and lopinavir-ritonavir (LPVr) and again, following rapid disease progression, 18 months after diagnosis with Efavirenz and Zidovudine (AZT) plus Lamivudine (3TC). Unbroken line with closed circles: CD4^+^ T-cell counts. Unbroken line with closed diamonds: Plasma viral loads. Broken line with closed circles: CD8^+^ T-cell counts. Blue arrow: negative HIV-1 antibody test. Red arrow: positive HIV-1 antibody test. Black arrows: Proctitis (1) / Shigellosis (2).

The HIV-1 patient tested Δ32/ Δ32 homozygous (termed patient #12 hereafter) is a German male in his early 40s whose only risk factor for HIV-1 infection is homosexual contact. Results of HIV-1 antibody / antigene tests were repeatedly negative until mid-February, 2002. In June 2002, this patient presented with weakness, a sore throat, pronounced cervical, inguinal and axillary lymphadenopathy, a truncal erythematous macular rash as well as leukopenia (nadir, 2.700 µl^−1^ ) and thrombocytopenia (79 µl^−1^). A suspect diagnosis of acute retroviral syndrome was corroborated by a positive enzyme immunoassay. Western Blot was initially negative for all bands, but turned positive for the p24 and GP160 bands on the patient's follow-up visit one week later. At this visit, his CD4^+^ T-cell count was 344 µl^−1^, his CD8^+^ T-cell count was 826 µl^−1^ and the viral RNA load in his plasma had a concentration of 2.323.000 copies ml^−1^. Highly active antiretroviral therapy (HAART) with efavirenz, stavudine and lopinavir-ritonavir was initiated 8 days after the first positive test result. Full suppression of viremia and a CD4^+^ T-cell count rise were observed until eight months after infection (Fig. 3), when treatment was suspended following the request of the patient. Clinically, apart from one episode of Chlamydia-associated proctitis successfully treated with azithromycine, this patient was doing well subsequent to HAART discontinuation. However, in the following months disease progression parameters deteriorated rapidly. Within the course of ten months CD4^+^ T-cells count dropped from 924 µl^−1^ (28.1%) to 275 µl^−1^ (12.9%), while plasma viral load rose from below the detection limit of 50 copies ml^−1^ to 22 400 copies ml^−1^. This prompted re-initiation of antiretroviral therapy with zidovudine plus lamivudine and efavirenz; viral load became undetectable and CD4^+^ T-cell counts recovered. A CD4 decline to 164 µl^−1^, seen when the patient suffered from Shigella sonnei gastroenteritis, was transient. As of now, patient #12 has remained on the same therapeutic regime for over four years, with stable CD4^+^ T-cell percentages ranging between 20 and 26% µl^−1^ and viral loads below the limit of detection.

## Discussion

### Distribution of CCR5Δ32 genotypes among seropositive and seronegative study participants

Variations within the host's genome contribute substantially to the individual course of HIV-1 infection. Our results obtained from the examination of a large study collective confirm the previous findings of a high prevalence of the CCR5Δ32 allele in European Caucasians [21], [38], [39]; it may therefore significantly affect susceptibility to HIV-infection, as well as progression to AIDS in this population.

Some studies have proposed that CCR5Δ32 heterozygotes could be protected against HIV transmission [6], [40], [41]. The seronegative controls examined here are not exposed but uninfected individuals in the strict sense. However, assuming that the resistance conferred by the +/Δ32 genotype was strong, our study would have revealed an under-representation of this genotype in HIV-infected as compared to uninfected subjects. As CCR5+/Δ32 frequencies were similar in both groups, our results are more in accordance with those described by other investigators, indicating that heterozygosity-associated protection is mild [1], [7].

### Association of CCR5Δ32 carriage with HIV-1 disease progression in Caucasian seroconverters

The well-established CCR5+/Δ32 related restriction against disease progression was also reflected in this survey. Seroconverters displaying the normal gene variant were compared with seroconverters displaying the deletion. The CCR5+/Δ32 genotype was related to lower setpoint viral loads, as well as slower progression to the study endpoint of 200 CD4^+^ cells^−µl^ in the Kaplan-Meier analysis [7]–[11], [41].

### Identification of an HIV-1 positive individual homozygous for the CCR5Δ32 deletion

So far, only 11 CCR5Δ32 homozygous HIV-1 infected individuals with the CCR5 Δ32/Δ32 genotype have been identified amongst a total of more than 20,000 HIV-positive individuals enrolled in several globally distributed cohorts. We here report one more of these extremely uncommon cases in a German study collective, further underscoring the fact that resistance conferred by the CCR5 Δ32/Δ32 genotype is strong but not complete. Transmission via contaminated blood products in hemophiliacs [23], [26] or by heterosexual contact [21] have been reported twice and once, respectively. In the majority of these cases [17]–[20], [22], [24], [25], and also in patient #12, transmission was through homosexual contact. However, this may well be due to the fact that this risk group is overrepresented in the cohorts studied so far.

Patient #12 has been under continuous observation since seroconversion and extensive clinical information is available. Under antiretroviral treatment, which was initiated twice in this individual, disease progression was satisfactorily controlled. However, a therapy-free period of 10 months revealed that the natural disease course in this patient would have been devastating. The apparently paradoxical association of the supposedly protective Δ32/Δ32 genotype with unusually rapid deterioration of immune status has been observed previously [22], [26] and implies that this genotype predisposes for rather pathogenic HIV variants; indeed, proviral DNA and viral RNA sequence analysis indicated CXCR4 tropism of the virus isolated from patient #12 (Kuecherer et al., separate manuscript in preparation). Exclusive CXCR4 tropism has been described in most [23], [24], [26], [42], but not all [19], [20] of the cases where the viral strain could be isolated. Presence of CXCR4-tropic HIV-1 strains has been associated with accelerated disease progression during antiretroviral therapy [43] as well as in natural disease studies [44]–[46].

Our findings included, 17% (i.e., 2 out of 12) of the known HIV-infected CCR5 Δ32 homozygotes are of German origin and have obtained infection in Germany. A common transmission chain seems rather unlikely to us, given the different geographical residences of the patients [Hannover (Patient#5, [21]), Berlin (Patient #12, this report)], as well as the different transmission routes involved [i.e. heterosexually [21], homosexually (this report)]. Neither we nor other German investigators [21], [39] have found a higher Δ32 allele frequency than that reported for most Western and Central European countries [1]. Far from being conclusive, our results might therefore indicate an enhanced circulation of CXCR4–tropic viral strains in this geographical region. With regard to the recent introduction of CCR5 antagonists as antiretroviral agents, this would be of paramount interest to the clinician.

## Materials and Methods

### Study population

The details of the study group, which comprised 737 HIV-1-positive adults and 463 seronegative controls, are provided in Table 2. Seropositive individuals are either enrolled in the *German HIV-1 Seroconverter Study* (n = 648) or were recruited prospectively in a private medical clinic (Jessen-Praxis, Berlin, Germany) (n = 89); written and informed consent has been obtained from all individuals.

**Table 2:** Demographic characteristics of HIV positive and HIV negative studied subjects

	HIV-positive	HIV-negative
Age^1^	39 (34–43)	38 (33–43)
	number (%)	
Ethnicity		
Caucasian	711 (96.5)	428 (92.4)
African	26 (3.5)	35 (7.6)
Gender		
Male	674 (91.5)	434 (93.7)
Female	63 (8.5)	29 (6.3)
Risk Group		n. d.
MSM	599 (81.3)	
Heterosexual	79 (10.7)	
IVDA	22 (3.0)	
HPL	21 (2.8)	
other^2^	16 (1.8)	
Total	737 (100.0)	463 (100.0)

1 Age median (IR, interquantile range) in years.

2 Of these, n = 2 individuals were occupationally exposed to HIV; risk group is unknown for n = 14 individuals.

The *German HIV-1 Seroconverter Study*, representing the German component of the CASCADE collaboration (*C*oncerted *A*ction on *S*ero*c*onversion to *A*IDS and *D*eath in *E*urope) [27]–[29], is a nationwide trial initiated by the Robert Koch-Institute, Berlin, in 1997. The study cohort has been described in considerable detail elsewhere [30]–[32]. Briefly, HIV-1 patients with either a documented HIV-antibody seroconversion (maximum time interval between last negative and first positive HIV-1 antibody test: 3 years) or documented ongoing seroconversion (i.e., HIV-RNA, antigen or antibody detection with an indeterminate immunoblot followed by completion of seroconversion) were recruited by the primary care physicians or clinical centers listed at the end of the manuscript. Documentation is subject to yearly follow-up and includes demographic and clinical information as well as CD4^+^ T-cell values, viral loads and antiretroviral therapy status. To examine the influence of host genetics on HIV-1 infection, genomic DNA of consenting patients has been prepared from peripheral blood specimens. 648 of the DNA samples were available for CCR5Δ32 genotype assessment. The median age in this patient group was 39 years (IR, 35–43 years) and 91% of the study participants were male. The four largest transmission groups are men having sex with men (MSM, 80%), heterosexuals (12%), patients from high-prevalence countries (HPL, 3%), and i.v. drug users (IVDA, 3%).

For the purpose of this and other studies on the influence of host genetic variation on susceptibility and progression of HIV-1 disease, we have been recruiting additional seroprevalents (i.e., the duration of infection is unknown) and seroconverters not participating in the German Seroconverter Study in a private German clinic since 2004, 89 of whom were genotyped for presence of the CCR5Δ32 deletion [33]. The median age in this collective is 37 years (IR, 33–40) and most (96%) of the study participants are European-Caucasian MSM. Demographic, laboratory and clinical data, as well as the current therapy status, are documented during the routine clinical visits (for the majority of participants, 2 – 4 times / year), and are considered retrospectively as well as prospectively. For genotyping, DNA was isolated from EDTA whole blood samples for all study participants.

The HIV-negative control collective consisted of 428 anonymized seronegative Caucasian blood donors, and 35 healthy, unrelated volunteers. All investigations have been conducted according to the principles expressed in the declaration of Helsinki; written approval has been granted from the Charité-Universitätsmedizin Berlin Ethical Board for all studied subjects.

For disease progression studies, only those CD4^+^ T cell and viral load values gathered before the start of any antiretroviral treatment were considered in order to preclude confounding effects. Disease outcome was defined either by viral setpoint (data available for 332 seroconverters, see Statistical Methods for definition) or, in survival analysis of 506 seroconverters, by analyzing the time from seroconversion to a CD4^+^ T cell count below 200 µl^−1^.

### CCR5Δ32 genotype assessment

CCR5Δ32 genotyping was performed by polymerase chain reaction (PCR) and subsequent gel electrophoresis. The reaction mix of 25 µl contained MgCl_2_ at a concentration of 1.5 mM (Qiagen, Hilden, Germany), dNTPs at 200 µM (Rapidozym, Berlin, Germany), 0.5 U HotStar Taq DNA polymerase (Qiagen, Hilden, Germany) and 5–20 ng DNA. Primers (forward, 5′-CTTCATCATCCTCCTGACAATCG-3′; reverse, 5′-GACCAGCCCCAAGTTGACTATC-3′; TibMolbiol, Berlin, Germany) were used at 0.5 µM each and have been previously described [34]. An initial denaturation step of 95°C for 15 min was followed by 40 amplification cycles [94°C, 30 s; 58°C, 30 s; 72°C, 45 s] and final extension at 72°C for 2 min. PCR amplicons of either 262 bp (CCR5Δ32 wildtype) or 230 bp length (CCR5Δ32 deletion) were visualized in a 3% ethidium bromide-stained agarose gel. Heterozygous samples yielded both a 262 bp and a 230 bp band. For the HIV-infected Δ32/Δ32 homozygous patient, presence of the deletion was verified by molecular sequencing and by testing DNA from a second blood sample collected on a separate occasion; furthermore, confirmatory PCR using a different primer set [35] was performed under the reaction conditions described above. Homozygous wildtype, homozygous mutant, and negative control samples were included in each reaction.

### Statistical methods

CCR5Δ32 genotype frequencies between seropositive and seronegative individuals were compared using the χ2 test; if expected frequencies were below 5, the exact version of the χ2 test was applied. Viral setpoints were defined as the median log10-transformed copies of viral RNA measured in plasma between 100 days and 2 years post infection [36], [37]. The strength of the association between CCR5Δ32 presence and disease severity was tested by comparison of the individual viral setpoints using the nonparametric Mann-Whitney test. For survival analysis, the Kaplan Meier method was applied, with the study endpoint defined as a CD4^+^ T-cell count of <200 cells µl^−1^. Differences between genotypes were tested by the log-rank test. We used SPSS14.01 for data management and statistical analyses, and PRISM 4 for figures.
